# The Book World of Medicine and Science

**Published:** 1900-06-30

**Authors:** 


### The Book World of Medicine and Science.

Report of the Malaria Expedition of the Liverpool School of Tropical Medicine and Medical Parasitology. By Donald Ross, D.P.H., M.R.C.S., H. E. Annett, M.D., D.P.H., and E. E. Austen, with Supplementary Reports by Major G. M. Giles, M.B., F.R.C.S., and R. Fielding-Ould, M.A., M.B. (Liverpool: The University Press. 1900. Price 10s. 6d. net.)

This is a very careful report of the work done by the expedition sent out to the West Coast of Africa by the Liverpool School of Tropical Medicine, with the object of investigating the natural history of malaria, and if possible devising some means for its prevention. But the book is something more than a mere report, for it contains a very complete review of the present position of our knowledge in regard to the origin and spread of this most important disease. We need hardly say that the drift of the report is to confirm very strongly the mosquito theory of the dissemination of the malaria, taking this theory in its neatest and most complete form, namely, that the malaria parasite, to go through the complete cycle of its life, must pass part of its time within the mosquito and another part in man, and that its transference from host to host takes place when the mosquito fills itself with the blood of man. The life history of the hæmamœbidæ is pictured as follows: Starting with the parasites in their youngest form as amœbulæ within the red corpuscles of the vertebrate hosts they, on reaching maturity, become either sporocytes or gametocytes. The sporocytes are destined for the sexual propagation of the organisms within the vertebrate hosts. By division, they give rise to spores which escape into the plasma, enter fresh corpuscles, and in their turn become amœbulæ again—thus, so far as the individual affected is concerned, continuing the species indefinitely in his blood. The gametocytes, however, are sexual forms, male and female. So long as they continue within their vertebrate hosts they remain unchanged; but on being drawn into the stomach of a suctorial insect they perform their sexual function, and the fertilised product is called a zygote. Again the process ends, unless this suctorial insect happens to be a species hospitable to the parasite. If, however, this insect should be one fitted to become a host to the parasite, the zygote pierces the wall of the stomach, and affixes itself in or on the outer coat, where it grows largely in bulk, its substance dividing into from eight to twelve meres. Each mere becomes a blastophore, bearing a large number of filamentous blasts affixed to its surface by one extremity. As growth advances the blastophores disappear, leaving the capsule of the zygote packed with the blasts. The capsule now ruptures, allowing the blasts to escape into the body cavity of the insect, whence they work their way into the insect's salivary gland, and finally pass from this gland into the blood of a new vertebrate host in which they eventually become the amœbulæ with which the cycle commenced. This, then, is the life history of the hæmamœbidæ, so far as it is at present known, and if for "vertebrate host" we substitute the word "man," and for "suctorial insect of a species hospitable to the parasite" we substitute the word "mosquito," we have the whole faith and creed of the mosquito theory of malaria.

Practical Gynæcology: A Handbook of the Disease of Women. By Heywood Smith, M.A., M.D., Oxon. Second Edition. (London: Henry J. Glaisher. 1900. 5s. net.)

More than twenty years ago Dr. Heywood Smith wrote the first edition of this little book, and now he issues to the world the second. We confess that we think he would have done better to let well alone. No one who has watched the development of gynæcology during the past twenty years can admit for a moment that the second edition of this little book shows an advance on the first corresponding in any degree with the changes which have taken place in the subject dealt with. The book is nicely printed, the subject is treated on a very systematic plan, and the type used to display the various headings under which the subject is dealt with is clear. But in regard to the matter itself we will only say that it does not appear to us to represent the gynæcology of to-day.

Diet and Food. By Alexander Haig, M.A., M.D., &c. (Illustrated. Pp. 97. Second edition. London: J. and A. Churchill. 1900.)

In the brief interval that has elapsed since the first edition of this work appeared Dr. Haig's theories have, in the judgment of many, received some shrewd knocks. But Dr. Haig confidently states that nothing has occurred to diminish his belief in the importance of the subject. Certainly the rapid call for a second edition is proof, if proof be needed, of what we remarked in reviewing the first edition—that Dr. Haig's practical directions, whatever be the value of the explanatory and much-debated theories with which they are prefaced, are good, and meet with the acceptance of many practitioners.

Nordrach at Home. By Jos. J. S. Lucas, B.A., M.R.C.S., L.R.C.P. (Pp. 60. 12mo. Bristol: J. W. Arrowsmith and Son. 1900. Price 1s.)

This brochure is neither better nor worse than the many dealing with the same subject with which the medical press is just now choked. The author, who has been Medical Registrar and Pathologist at the North London Consumption Hospital, writes pleasantly and simply of the hygienic treatment of consumption as adapted to English home life. Internal evidence makes us believe that this little work has been written for popular instruction rather than for medical men. It can do no harm, and may do good. But, as the author himself observes, there is nothing very original in it.

### NOTES ON BOOKS.

We are glad to welcome the new edition of B. Bradshaw's "Dictionary of Bathing Places and Climatic Health Resorts, 1900" (Kegan Paul, Trench, Trübner, and Co.) It is handy in form and small in size, yet the number of places mentioned in it is enormous, and in view of the frequency with which medical men are apt to be consulted in regard to out-of-the-way health resorts it will be found a very useful book for the consulting-room table.

### BOOKS RECEIVED.

Kegan Paul, Trench, Trübner, and Co.

"B. Bradshaw's Dictionary of Bathing Places and Climatic Health Resorts."

